# Elevated TRIM44 promotes intrahepatic cholangiocarcinoma progression by inducing cell EMT via MAPK signaling

**DOI:** 10.1002/cam4.1313

**Published:** 2018-02-15

**Authors:** Rui Peng, Peng‐Fei Zhang, Chi Zhang, Xiao‐Yong Huang, Yan‐bing Ding, Bin Deng, Dou‐Sheng Bai, Ya‐Ping Xu

**Affiliations:** ^1^ Department of Gastroenterology Shanghai Tenth People's Hospital Tongji University School of Medicine Shanghai 200032 China; ^2^ Department of Hepatobiliary and Pancreatic Surgery Subei People's Hospital Clinical Medical School Yangzhou University Affiliated Hospital Yangzhou China; ^3^ The Second Affiliated Hospital of Xiangya School of Medicine Central South University Hunan China; ^4^ Department of Oncology Shanghai East Hospital Tongji University School of Medicine Shanghai China; ^5^ Liver Cancer Institute Ministry of Education Zhongshan Hospital Fudan University Key Laboratory of Carcinogenesis and Cancer Invasion (Fudan University) Shanghai 200032 China; ^6^ Department of Gastroenterology Yangzhou No. 1, People's Hospital The Second Clinical School of Yangzhou University Yangzhou China

**Keywords:** Apoptosis, EMT, ICC, prognosis, TRIM44

## Abstract

Surgical results for intrahepatic cholangiocarcinoma (ICC) remain unsatisfactory due to the high rate of recurrence. Here, we investigated that the expression and roles of tripartite motif‐containing protein 44 (TRIM44) in human ICCs. Firstly, TRIM44 expression was analyzed in several kinds of cancers by referring to public Oncomine database, and the expressions of TRIM44 mRNA and protein were tested in ICC and corresponding paratumorous tissues. Secondly, functions and mechanisms of TRIM44 in ICC cells were further evaluated by TRIM44 interference and cDNA transfection. Finally, the prognostic role of TRIM44 was assessed by Kaplan–Meier and Cox regression. We found that TRIM44 expression was upregulated in ICC tissues compared with corresponding paratumorous tissues, which were consistent with the results from the public cancer database. Knockdown of TRIM44 repressed the invasion and migration of ICC cells, while increased the ICC cell apoptosis. Additionally, high level of TRIM44 was shown to induce ICC cell epithelial to mesenchymal transition (EMT). Mechanistically, a high level of TRIM44 was found to activate MAPK signaling, and a MEK inhibitor, AZD6244, reversed cell EMT and apoptosis endowed by TRIM44 overexpression. Clinically, TRIM44 expression was positively associated with large tumor size (*P* = 0.035), lymphatic metastasis (*P* = 0.008) and poor tumor differentiation (*P* = 0.036). Importantly, patients in TRIM44^high^ group had shorter overall survival and higher cumulative rate of recurrence than patients in TRIM44^low^ group. Our results suggest elevated TRIM44 promotes ICC development by inducing cell EMT and apoptosis resistance, and TRIM44 is a valuable prognostic biomarker and promising therapeutic target of ICC.

## Introduction

Intrahepatic cholangiocarcinoma (ICC) is a malignant epithelial neoplasm derived from primary and secondary bile tracts, and accounts for 5–10% primary liver cancer (PLC) 1. Now, ICC patients who underwent surgical treatment or curative liver transplantation still have highly recurrence rates due to its prone to microvascular invasion and lymphatic metastasis at the tumor early stage 2. Unfortunately, the mechanism of the ICC recurrence is still in limited understanding 3. Thus, it is urgently to disclose the mechanism of ICC progression which may help to develop effectively therapy strategies for ICC.

Triple motif (TRIM) family proteins, contain three conserved domains, including RING finger, B‐box, and coiled‐coil. TRIMs were reported to be involved in a variety of biological processes, including cancer 4. For example, TRIM19, TRIM24 and TRIM25 had been revealed to be influential regulators for tumorigenesis 5, 6. As an important member of TRIM family, TRIM44's N‐terminal region had a zinc‐finger domain, which possess the activity of ubiquitin‐specific proteases (USPs). Thus it was defined to be a new class of the “USP‐like TRIM” 7, many of which has been unveiled to be involved in cancer pathogenesis, for example, USP7 was recently uncovered to promote the progression of hepatocellular carcinoma (HCC) 8. Previous studies have demonstrated that TRIM44 was responsible for multiple disorders such as neurodegenerative diseases and viral infections 9. Recently, TRIM44 was reported to play a cancer‐promoting role in a variety of cancers, including head and neck squamous cell carcinomas and esophago‐gastric cancer 10, 11. Importantly, the latest studies suggested that high level of TRIM44 induced cancer cell epithelial to mesenchymal transition (EMT) and participated in tumor initiation and progression by activating PI3K/AKT/mTOR pathway 12, 13. Additionally, another report demonstrated that TRIM44 facilitated the migration and invasion of cancer cells via activating the NF‐κB pathway in lung cancer 14. Indeed, gene set enrichment analysis showed that genes related to TRIM44 expression were definitely enriched in invasion, migration, proliferation and cell apoptosis 12, 15. Thus, TRIM44 appears to be a vital promoter in tumor development. However, the expression and molecular pathogenesis of TRIM44 in ICC remain unclear.

In this study, we analyzed the level of TRIM44 in human ICCs, and tried to uncover the roles and mechanisms of TRIM44 in ICC cell proliferation, invasion and apoptosis. Finally, the clinical implication of TRIM44 in ICC patients was further determined.

## Materials and Methods

### Patients and specimens

Thirty‐two pairs of frozen tumor and matched peritumor samples randomly collected from the tissue bank at the Subei People's Hospital of Yangzhou University were analyzed by western blotting and quantitative real‐time polymerase chain reaction (qRT‐PCR), and 8 pairs of samples were selected for immunohistochemistry (IHC). A total of 130 paraffin‐embedded ICC tumors and matched peritumor tissues were collected to construct tissue microarray (TMA) as previous report 16. The clinical data and prognosis were collected from 2007 to 2012. The study obtained the written informed consent from each patient with the Subei People's Hospital of Yangzhou University Research Ethics Committees' permission.

### Tissue microarrays and IHC

Tissue microarray was constructed as described 8. The procedures of IHC for TRIM44, E‐cadherin, Vimentin, *β*‐catenin, Snail were performed in the early studies 17, 18. The IHC staining and positive criteria were done as previously described 17, 19. The antibodies' information was listed in Table S1. The density level of TRIM44 was defined by the intensity and percentage of positive staining with cytoplasm in the whole cylinder as described 16 and in the Data S1. The percentage of TRIM44‐positive cells was scored in five groups: 0 (0%), 1 (1 to ≤25%), 2 (25 to ≤50%), 3 (50 to ≤75%) and 4 (>75%). The 0, 1, and 2 groups defined as low expression, while 3 and 4 groups defined as high expression.

### Cell cultures

The human ICC cell lines RBE and QBC939 were purchased from the Chinese Academy of Science Cell Bank (Shanghai, China) as our previous study 20. Cell lines were routinely checked for contamination by *Mycoplasma*, using Hoescht staining, and were authenticated by DNA‐Fingerprinting and isoenzyme analyses. These cell lines were obtained within 6 months before being used for this study. Both cell lines were maintained in RPMI1640 (Gibco, USA) supplemented with 10% fetal bovine serum (Gibco, USA) and 1% penicillin/streptomycin (Corning, Lowell, MA) at 37°C in a humidified incubator with 5% CO_2_.

### Western bolting and qRT‐PCR

Western blotting were performed as previous research 19. And the primary antibodies' details were listed in Table S1. The total RNA from cell lines and frozen tissues was extracted by TRIzol reagent (Invitrogen, USA), and reversed to cDNA by prime Script RT reagent kit (Takara, Japan). Primers for PCR were displayed in Table S2. All experiments were performed in triplicate.

### Transfection of lentiviral vectors with shRNA of TRIM44

pGMLV‐SC5‐Puromycin‐EGFP‐shRNA‐TRIM44 and pGMLV‐PE3‐RFP‐TRIM44 lentiviral vectors were purchased from Shanghai Genomeditech company (Shanghai, China). The shRNA1 sequence is GCCTTTGAAGAATTAAGAAGC and the shRNA 2 sequence is GCAGA AGGCCCTTCATCTAGT. The TRIM44‐shRNA vectors were transfected into QBC939 cells and pGMLV‐SC5‐Puromycin vectors were used as control. The pGMLV‐PE3‐TRIM44 lentiviral vectors were transfected into RBE cell line. Stably transfected cells were confirmed by western blot and qRT‐PCR.

### Immunofluorescence assays

After treatment with 0.1% Triton X‐100 for 30 min at 25°C, cells were washed with PBS, and then blocked with 10% bovine serum albumin and incubated with the primary antibodies overnight at 4°C. Following washing with PBS in triplicate, cells were incubated with secondary antibodies for 2 h. Finally, cell nuclei were stained by diamidino phenylidole (DAPI) and photographed by the fluorescence microscope (Olympus, Japan).

### CCK8 assays, wound healing assays and invasion assays

For CCK8, it was performed as previous research 21. For wound healing assay, the wound lines were scratched by 200 *μ*L pipette tip until cells covered 95% of the 6‐well plated bottom. Then migrating cells were measured under a microscope at 0 and 72 h. For cell invasion assays, cells were incubated in 24‐well transwell precoated with matrigel (Falcon354480; BD Biosciences, USA). A total number of 2 × 10^4^ cells resuspended in 200 *μ*L RPIM‐1640 were added upper chambers, and 600 *μ*L RPIM‐1640 with 10% FBS were added lower chambers. The transwell was incubated with 5% CO_2_ at 37°C for 48 h, then cells were fixed in 4% paraformaldehyde and stained by crystal violet. Cells in 5 random fields (magnification, ×100) were counted and photographed. All results are mean ± SD of 3 independent experiments.

### Flow cytometry assays

Flow cytometry assay (FACS) was utilized to analyze cell apoptosis. A total number of 1 × 10^5^ cells were collected to centrifugal tube and stained by Annexin V‐APC/7‐ADD Apoptosis Detection Kit (Yeasen, China). All experiments were repeated for three times.

### Statistical analysis

Statistical analyses were performed using SPSS 21.0 software. Student's *t*‐tests was adopted to analyze quantitative variables. Kaplan–Meier analysis was utilized to analyze survival. Survival curves were estimated using a log‐rank test. The cox proportional hazards model was used to assess the univariate or multivariate hazards. The *P* value <0.05 was regarded as statistically significant.

## Result

### TRIM44 expresses highly in several human digestive cancers and ICC tissues

**Figure 1:**
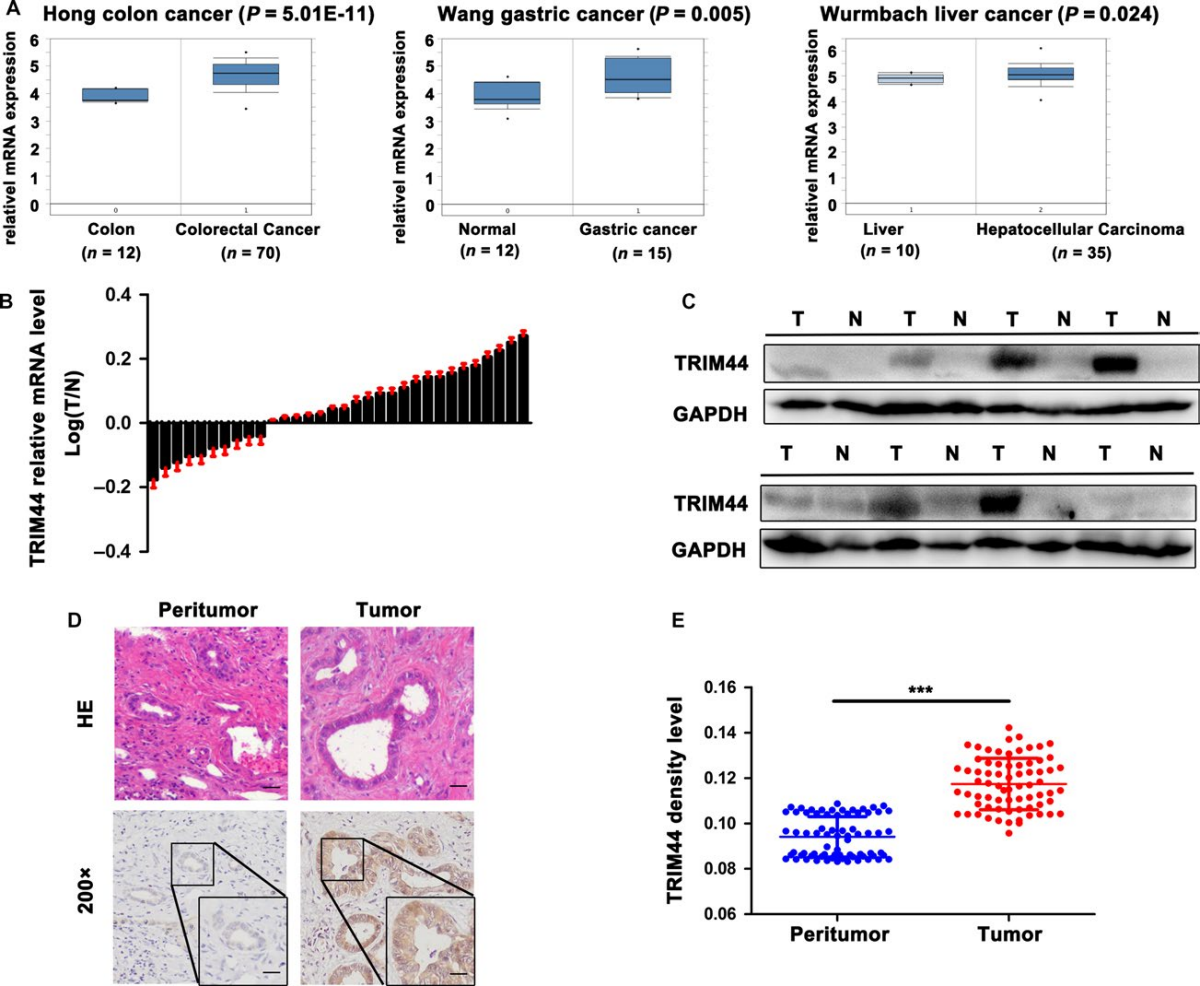
Expression of TRIM44 in human cancer and ICC. (A) Microarray data analyses from the oncomine database presented that TRIM44 mRNA expression in colon cancer, gastric cancer and liver cancer, and the TRIM44 were increased in cancer compared with their normal tissues, which was conducted using the oncomine software. The boxes represent the 25th through 75th percentiles. The horizontal lines represent the medians. The whiskers represent the 10th and 90th percentiles, and the asterisks represent the end of the ranges. (B) The mRNA expression of TRIM44 in 32 paired ICC tumor and paired paratumor tissues. (C) TRIM44 protein level in patients tissues. (D) Representative HE and IHC graphs of TRIM44 in tumor and normal tissues. (E) Density analysis indicated that significant difference of TRIM44 between 130 ICC patients tumor and their normal bile duct tissues. Scale bar 200× and 50 *μ*m, 400× and 25 *μ*m *P < 0.05, **P < 0.01, ***P < 0.001.

Firstly, we analyzed the level of TRIM44 in three human digestive cancers from the Oncomine database, which contains cDNA microarray data for cancer and matched normal tissues. Several representative data were shown in Figure 1A, which indicated TRIM44 mRNA generally increased in colorectal cancer 22, gastric cancer 23 and HCC compared with their normal tissues 24. Thus, TRIM44 is up‐regulated in multiple human digestive cancer tissues.

Next, the TRIM44 mRNA was found to increase in 22/32 ICC tissues compared with the paratumorous normal tissues (*P* < 0.001, Fig. 1B). Western bolting indicated that the expression of TRIM44 was remarkably higher in ICC than that in adjacent tumor tissues (3.370 ± 2.314 vs. 1.249 ± 1.118, *P* = 0.0156, Fig. 1C). Histochemically, the positive staining of TRIM44 mainly localized in the cell cytoplasm, and the intensity of TRIM44 in tumor cells is higher than that in peritumor cells (Fig. 1D). Density analysis indicated 71 ICC tissues showed higher expression of TRIM44 than those in paired peritumor tissues (*P* < 0.001, Fig. 1E). Our results indicate that the levels of TRIM44 mRNA and protein are consistently increased in human ICC.

### TRIM44 promoted the invasion and migration of ICC cells in vitro

**Figure 2:**
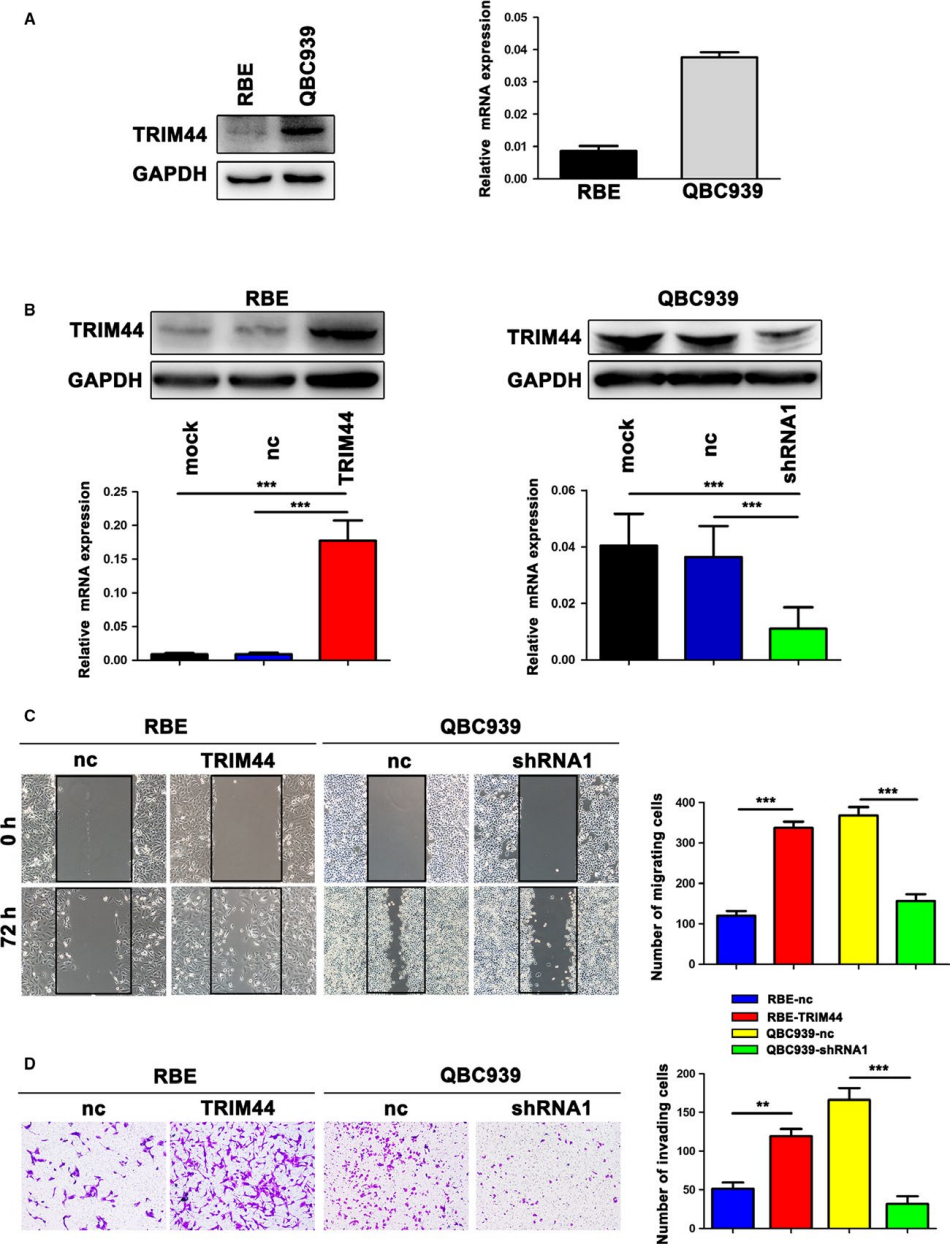
TRIM44 promotes ICC cell migration and invasion in vitro. (A) Relative expression of TRIM44 in RBE and QBC939 cell lines. (B) Efficiency of TRIM44 overexpression and inhibition were analyzed in ICC cell lines using western bolt and qRT‐PCR. (C) Wound healing assays showed the ability of TRIM44 in cell migration. (D) The invasion of four cells lines were measured by transwell assays. Data shown were means (±SD) from three independent experiments. *P < 0.05, **P < 0.01, ***P < 0.001.

Then, we chose two ICC cell lines to analyze the expression of TRIM44 (Fig. 2A). The RBE cell line, which expressed low level of TRIM44, was transfected with TRIM44 cDNA vectors and its control, while the QBC939 was transfected with TRIM44‐shRNA1 and the control lentivirus. Western bolting and qRT‐PCR were used to confirm the successful overexpression and knockdown of TRIM44 (Fig. 2B and Fig. S1A). We found that elevated TRIM44 expression enhanced RBE cells' ability of invasion and migration, and TRIM44 depletion remarkably inhibited the invasion and migratory of QBC939 cells (Fig. 2C and D and Fig. S1B and C). Collectively, these assays data show that TRIM44 serves as a promoter of ICC cells aggressiveness.

### Elevated TRIM44 appear to induce ICC cells apoptosis resistance

**Figure 3:**
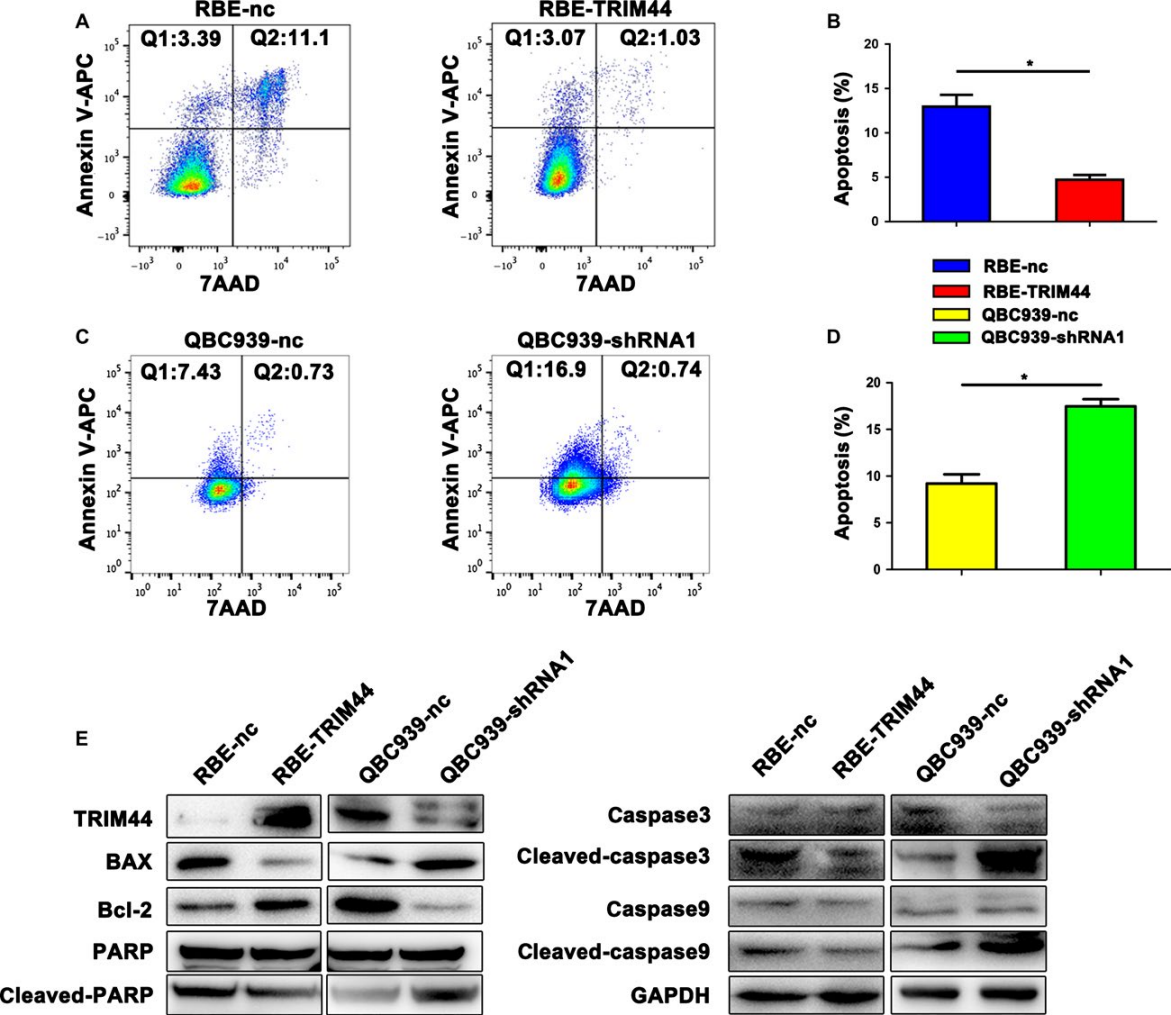
TRIM44 induced ICC cell apoptosis. (A and B) RBE‐TRIM44 and its control cells apoptosis were analyzed by FACS for annexin‐V APC/7AAD assays. (C and D) QBC939‐shRNA and its control cells apoptosis were analyzed by FACS for annexin‐V APC/7AAD assays. (E) Western blot indicated that TRIM44 induced the level of Bax, Bcl‐2, caspase3, caspase9, and PARP. GAPDH was used as internal control. The quadrant of Q1 means early apoptotic cells subpopulations. The quadrant of Q2 means late stage apoptotic cells subpopulations. The results represent the mean ± SD of three independent experiments. *P < 0.05, **P < 0.01, ***P < 0.001.

Here, we further employed the FACS to assess the role of TRIM44 in cell apoptosis. We found that TRIM44 overexpression resulted in a downregulated the percentage of apoptosis cells compared with the control cells (*P* = 0.03, Fig. 3A and B and Fig. S1D), whereas knockdown of TRIM44 definitely increased the rate of apoptosis (*P* = 0.012, Fig. 3C and D). Moreover, overexpression of TRIM44 was identified to be associated with downregulation of Bax and several caspase family proteins and up‐regulation of Bcl‐2 (Fig. 3E and Fig. S1E). The ICC cells viability was improved by high level of TRIM44 (Fig. S1F). These data suggest that TRIM44 may play a vital role in antiapoptosis.

### TRIM44 regulates ICC cells invasion via EMT

**Figure 4:**
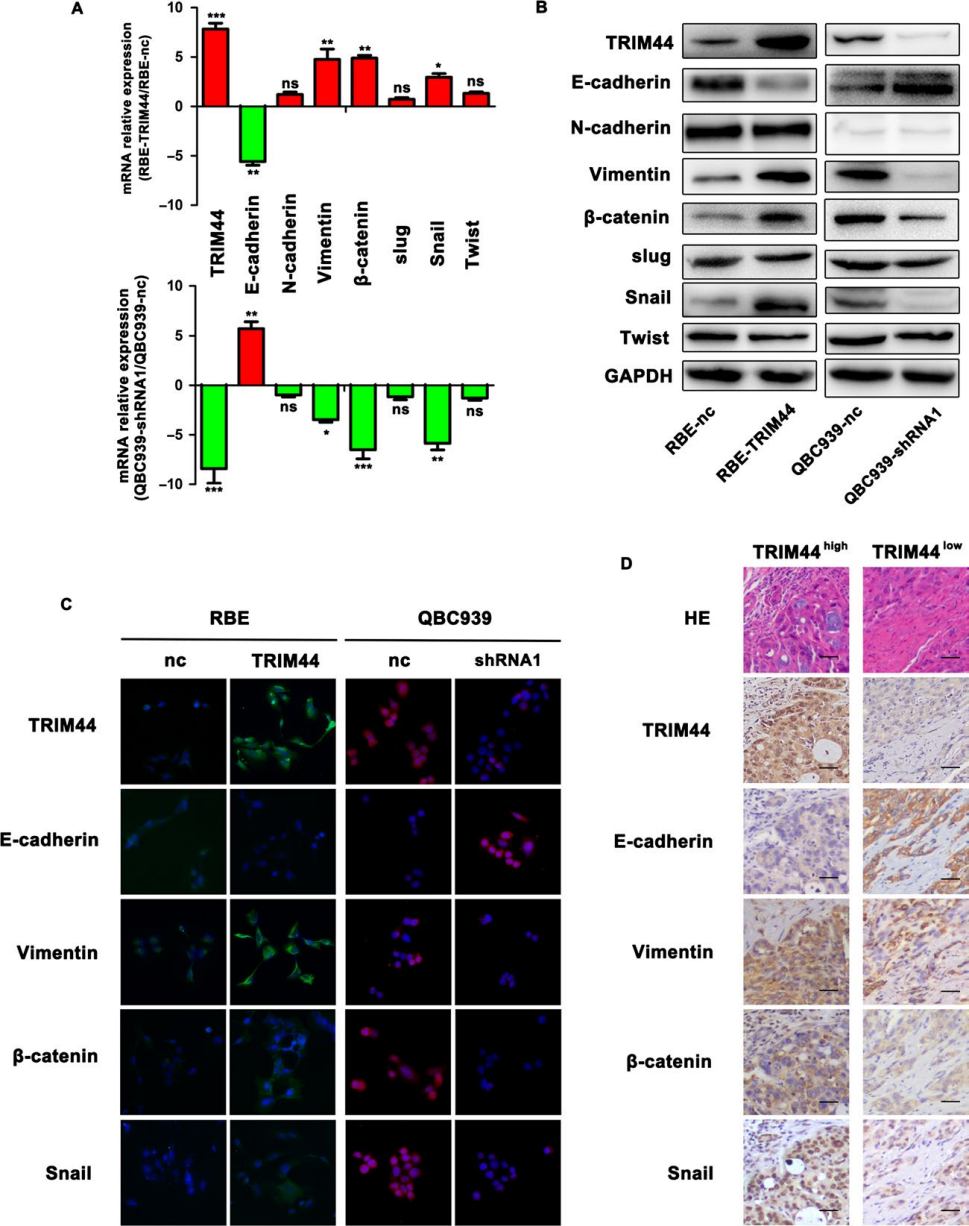
Overexpressed TRIM44 promotes ICC cell invasiveness by enhancing EMT. (A and B) The mRNA and protein of EMT factors was compared between four ICC cell lines (RBE‐nc, RBE‐TRIM44, QBC939‐nc, QBC939‐shRNA). (C) Immunofluorescent images of TRIM44, E‐cadherin, vimentin, *β*‐catenin and snail in four ICC cell lines are shown. (D) Representative IHC images of patients are shown. HE, hematoxylin and eosin. Of one patients with ICC showed TRIM44^high^, E‐cadherin^low^, and vimentin^high^, *β*‐catenin^high^, and snail^high^. Another exhibited TRIM44^low^, E‐cadherin^high^, whereas vimentin^low^, *β*‐catenin^low^ and snail^low^. Scale bar 200×, 50 *μ*m, *P < 0.05, **P < 0.01, ***P < 0.001.

Previous researchers have demonstrated TRIM44 can promote EMT in lung cancer and HCC 13, 25. Thus, we tried to investigate several EMT markers, including E‐cadherin, N‐cadherin, vimentin, *β*‐catenin as well as the EMT transcription factors snail, slug and twist, in the above four cell lines. The qRT‐PCR and Western bolting showed that E‐cadherin level was clearly decreased in RBE‐TRIM44 and QBC939‐nc, whereas vimentin, *β*‐catenin and snail were significantly increased in these two cell lines. However, there was no obviously alteration of N‐cadherin, slug, and twist expression (Fig. 4A and B and Fig. S2A and C), which were further ensured by immunofluorescence (Fig. 4C and Fig. S2B). Likewise, these markers were also confirmed by immunohistochemical staining in serial sections of ICC tissues, and in the positive TRIM44 staining of ICC tissues, downregulaton of E‐cadherin and up‐regulation of vimentin, *β*‐catenin and snail were observed in invasive tumor fringe and vice versa (Fig. 4D). These results indicate that TRIM44 overexpression can induce ICC cell EMT.

### TRIM44 regulated ICC cell EMT via MAPK signaling pathway

**Figure 5:**
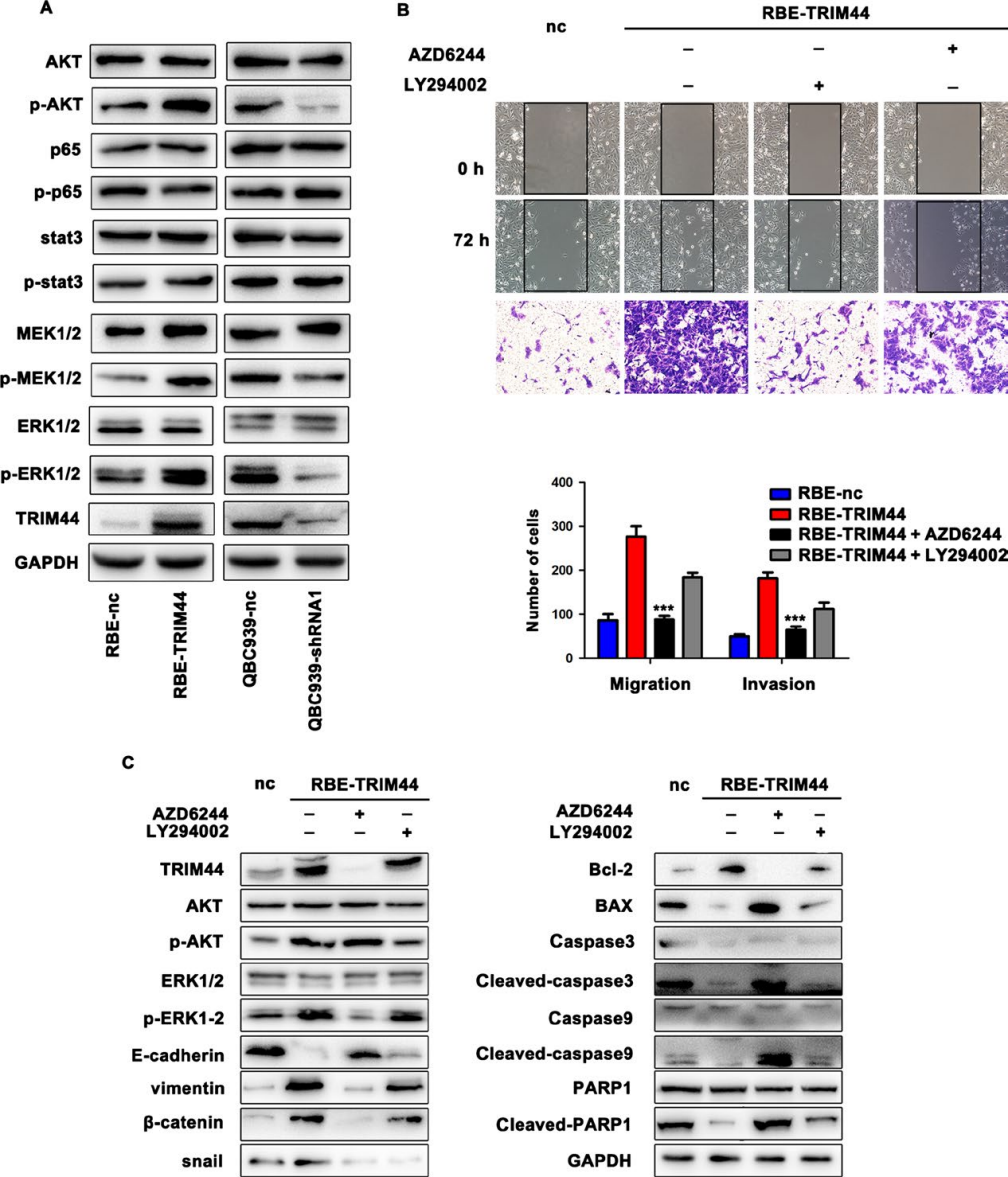
High level of TRIM44 induced the cells apoptosis resistance and EMT via MAPK pathway. (A) Several signal moleculars were observed in RBE‐TRIM44, QBC939‐shRNA and their control cells. (B) RBE‐TRIM44 was treated by AZD6244 (MEK inhibitor, 5 *μ*mol/L, 24 h) and applied to wound healing and invasion assays compared with RBE‐nc and RBE‐TRIM44 without AZD6244. (C) Western blot bands of TRIM44, ERK1/2, p‐ERK1/2, EMT markers and transcription factors were shown. GAPDH was used as internal control.

Here, we further determined the molecular mechanism involved in the TRIM44 functions, and we chose several acknowledged signal pathways in ICC progression to determine by western blot. The results showed p‐AKT expression was up‐regulated in RBE‐TRIM44 cells compared with control cells, while p‐AKT was repressed in QBC939‐shRNA cells compared with the QBC939‐nc cells. Interestingly, we found overexpression of TRIM44 in RBE cells could also up‐regulate phosphorylation of MEK and phosphorylation of ERK1/2 (Fig. 5A, and Fig. S3A), both of which belong to the MAPK pathway and is always aberrantly elevated in human cancers 26, 27. However, the p65, p‐p65, stat3, and p‐stat3 were not obviously influenced by TRIM44 expression in ICC cells (Fig. 5A). To examine whether a high level of TRIM44 influence tumor invasive and metastasis by MAPK pathway, we treated RBE‐TRIM44 with AZD6244, a MEK inhibitor (5 *μ*mol/L, 24 h). As presented in Figure 5B and Figure S3B, the AZD6244 prominently inhibited aggressiveness and metastasis of ICC cells with high level of TRIM44. Moreover, the treatment of AZD6244 not only induced upregulation of E‐cadherin, *β*‐catenin, and Bax, but also downregulated vimentin, snail and Bcl‐2 in RBE‐TRIM44 (Fig. 5C and Fig. S3C and D). These results suggest that high level of TRIM44 induces ICC cell EMT and apoptosis inhibition via MAPK pathway mainly.

### High level of TRIM44 is associated with poor prognosis of ICC patients

**Figure 6:**
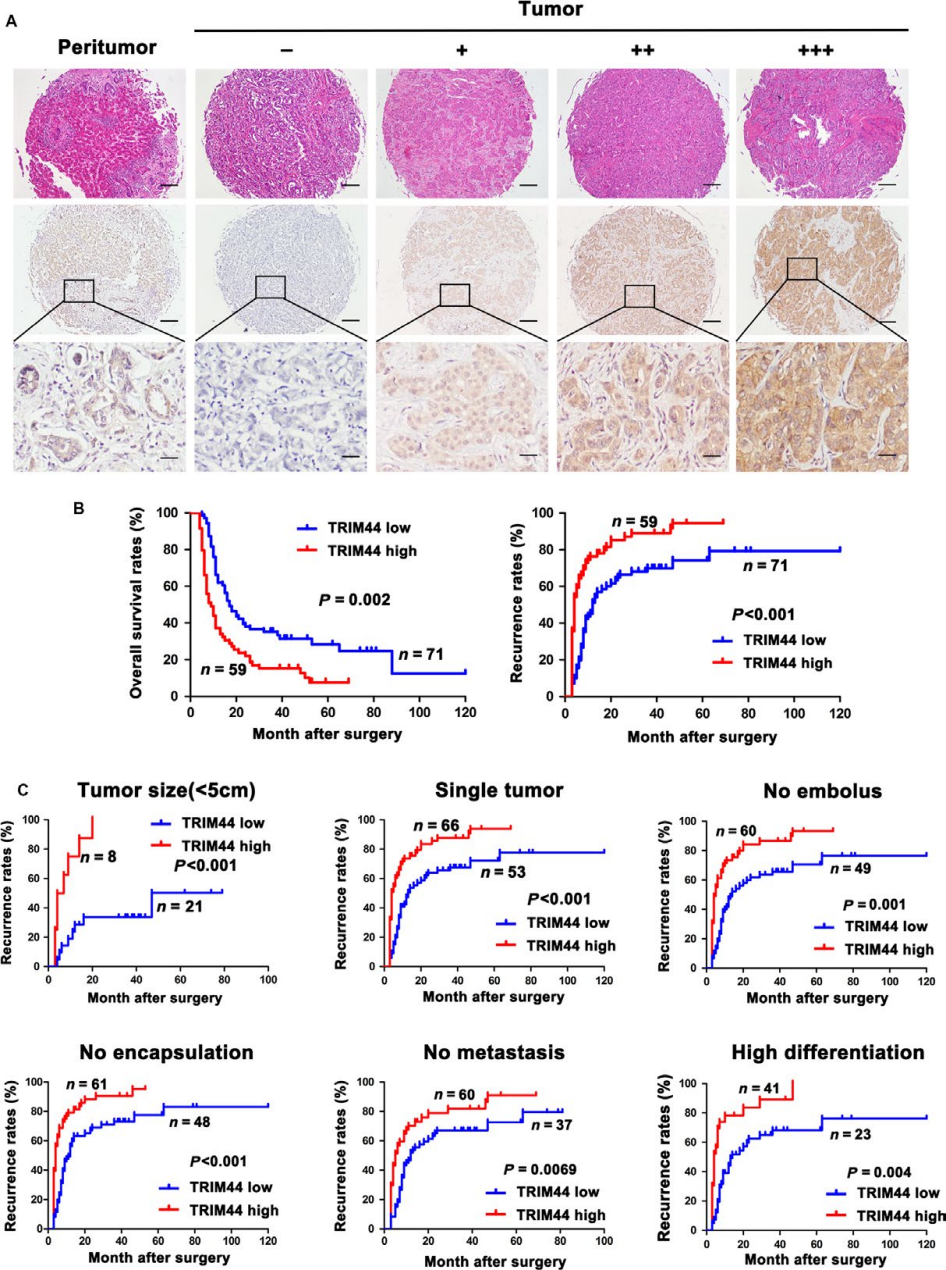
The expression of TRIM44 and its prognosis value in 130 ICC patients.(A) Representative images of tumor tissues in different staining were shown and graded from ‘−' to ‘+++'. Scale bar 40×, 100 *μ*m, 200×, 50 *μ*m. (B) Kaplan–Meier analysis of Overall survival and cumulative recurrence for TRIM44 expression. (C) prognostic value of TRIM44 in tumor size, single tumor, no embolus, no encapsulation, no metastasis, high differentiation subgroups.

We revealed that TRIM44 protein was incredible heterogeneity in tumor samples, and representative images were shown in Figure 6A. The weak TRIM44 staining was found in the normal biliary specimens, whereas variously level of TRIM44 was detected in tumor tissues (−, absent; +, weak; ++, moderate; +++, strong).

Furthermore, we found that TRIM44 expression was positively related to large tumor size (*P* = 0.035), lymphatic metastasis (*P* = 0.008), and poor tumor differentiation (*P* = 0.036, Table 1). By the end of following, 104 (80%) patients died. In total, the 2 and 5 years survival rates were 32% and 21%, respectively. And the group of TRIM44^high^ had lower rates of overall survival (OS) than that of TRIM44^low^. Moreover, the patients with TRIM44^high^ had significantly higher recurrence rates than those patients with TRIM44^low^ (*P* = 0.001 and *P* < 0.001, respectively, Fig. 6B). To better understand the prognostic value of TRIM44 in ICC patients, we further analyzed by dividing all the patients into several subgroups, and observed that predictive value of TRIM44 were continued in tumor size (<5 cm, *P* < 0.001), single tumor (*P* < 0.001), no embolus (*P* = 0.001), no encapsulation (*P* < 0.001), no metastasis (*P* = 0.0069), and high differentiation subgroups (*P* = 0.004; Fig. 6C).

**Table 1:** Correlations between TRIM44 with clinicopathologic features in 130 ICC patients

Variable	Number of patients		*P* value^a^
	TRIM44^high^	TRIM44^low^	
Age, y			
≥53	33	35	0.484
<53	26	36	
Sex			
Men	26	30	0.860
Women	33	41	
HBsAg			
Positive	36	45	0.856
Negtive	23	26	
Child‐Pugh score			
A	4	2	0.410^b^
B and C	55	69	
Serum CA19‐9, ng/mL			
≥37	41	39	0.105
<37	28	32	
Serum ALT, U/L			
≥75	9	10	1.000
<75	50	61	
SerumAFP, ng/mL			
≥20	9	6	0.276
<20	50	65	
Cirrosis			
Yes	20	33	0.157
No	39	38	
Tumor encapsulation			
Yes	11	10	0.633
No	48	61	
Maximal tumour size (diameter, cm)			
≥5	51	50	**0.035**
<5	8	21	
Tumor number			
Multiple	6	5	0.545
Solitary	53	66	
Embolus			
Yes	10	11	1.000
No	49	60	
Lymphatic metastasis			
Yes	22	11	**0.008**
No	37	60	
Tumour differentiation			
III/IV	36	30	**0.036**
I/II	23	41	

HBsAg, hepatitis B surface antigen; CA 19‐9, carbohydrate antigen 19‐9; ALT, alanine aminotransferase; AFP a‐fetoprotein.

a *P *< 0.05 was considered significant.

b Fisher exact test. Statistically signifcant values are shown in bold.

Univariate analysis showed that AFP, tumor number, lymphatic metastasis, tumor size, embolus as well as TRIM44 staining were associated with OS and cumulative recurrence. Moreover, tumor size, embolus were related to both OS and cumulative recurrence (Table 2). Thus, TRIM44 is a risk marker of the OS and the cumulative recurrence of ICC patients.

**Table 2:** Univariate and multivariate analyses of factors associated with recurrence and survival

	Overall survival				Cumulative recurrence			
	Multivariate analysis				Multivariate analysis			
Variable	Univariate *P*	HR	95% CI	*P* a	Univariate *P*	HR	95% CI	*P*
Age, y (≥53 vs. <53)	0.554			NA	0.393			NA
Sex (Men vs. Women)	0.589			NA	0.770			NA
HBsAg (Positive vs. Negative)	0.645			NA	0.811			NA
Child‐Pugh score (A vs. B)	0.173			NA	0.108			NA
Serum CA19‐9, ng/mL (≥37 vs. <37)	0.492			NA	0.404			NA
Serum ALT, U/L (≥75 vs. <75)	0.408			NA	0.824			NA
Serum AFP, ng/mL (≥20 vs. <20)	0.001			NS	0.048			NS
Cirrosis	0.365			NA	0.575			NA
(Yes vs. No)								
Tumor encapsulation (Yes vs. No)	0.254			NA	0.109			NA
Tumor differentiation (III/IV vs. I/II)	0.220			NA	0.215			NA
Tumor number (Multiple vs. Solitary)	0.016			NS	0.003			NS
Lymphatic metastasis (Yes vs. No)	0.037			NS	0.010			NS
Maximal tumor size (diameter, cm) (≥5 vs. <5)	0.005	0.551	0.318–0.957	0.034	0.002	0.504	0.291–0.874	0.015
Embolus (Yes vs. No)	0.006	0.501	0.306–0.822	0.006	0.013	0.504	0.307–0.828	0.007
TRIM44 staining (<50% vs. ≥50%)	<0.001	0.005	1.195–2.665	0.005	<0.001	1.936	1.296–2.890	0.001

HR, hazard ratio; CI, confidence interval; HBsAg, hepatitis B surface antigen; ALT, alanine aminotransferase; CA 19‐9, carbohydrate antigen 19‐9; AFP, a‐fetoprotein; NS, not significant; NA, not adopt.

a *P* < 0.05 was regarded as statistically significant, *P* value was calculated using Cox proportional hazards regression.

## Discussion

In this study, our results revealed that TRIM44 is crucial for the invasion and apoptosis of ICC cells in vitro. Moreover, we found that not only TRIM44 could increase the activation of AKT signaling pathway as previous reports 12, 13, but also activate ERK1/2, and the activation of ERK1/2 is responsible for the ICC cell EMT. Importantly, we showed the ICC patients with high level of TRIM44 had shorter OS than those with low level of TRIM44. These data imply that TRIM44 promotes ICC cell EMT via ERK‐MAPK pathway, and can serve as a biomarker of poor prognosis for ICC patients.

TRIM44 plays a significantly regulatory role in extensively biological processes, including cell proliferation, innate immunity, virus infection, and tumor development 4, 9. Here, we firstly showed that the level of TRIM44 mRNA was up‐regulation in several human digestive cancers according to a public database. Then overexpression TRIM44 in ICC tissues was clearly defined by qRT‐PCR and western blot, which were similar to previous studies in other cancers 11, 28. An important finding is elevated TRIM44 expression resisted to cell apoptosis. Previous studies demonstrated that decreased TRIM44 inhibited cell cycle through deregulating cyclins and CDKs 13, 25. Furthermore, overexpression of TRIM44 was reported to be associated with apoptosis inhibition in esophageal cancer 12. Meanwhile, microarray analysis showed that TRIM44 knockdown was associated with the dysregulation of NUPR1, CDK19, CADM1, INHBA, TNFSF10, and DDIT4, which could induce cell apoptotic 29. Interestingly, overexpression of TRIM44 significantly decreased Bax expression and enhanced expression of Bcl‐2, two key apoptosis regulatory factors 30, 31. Thus, TRIM44 played a vital role in inhibited cell apoptosis.

As acknowledged, EMT contributes to malignant tumors development 32. Our result revealed that the inhibition of TRIM44 remarkably up‐regulated the expression of epithelial marker E‐cadherin and decreased mesenchymal marker vimentin. Additionally, other EMT markers *β*‐catenin and transcription factors snail were apparently increased by forced TRIM44 expression. Previously study has noted that TRIM44 positively related to the activation of AKT/mTOR signaling pathway 12. And further evidences have indicated that overexpression of TRIM44 promoted cells resistance to doxorubicin via accelerating NF‐κB activation 14, 25. Similarly, our results indicated that overexpression of TRIM44 could influence phosphor‐AKT. Interestingly, we also found the up‐regulation of TRIM44 induced phosphorylation of ERK1/2. By using the AKT and MAPK signal inhibitor, we found that the inhibition of MAPK signal significantly repression ICC cell invasion and metastasis endowed by TRIM44. Importantly, the EMT phenotype of cells overexpressed TRIM44 was also reversed by MEK1/2 inhibitor, while partially reversed by the AKT inhibition. It is had been illustrated that the activation of MAPK signal pathways induced several cancer cells EMT 20, 26, 33, 34. Thus, we conclude that the MAPK pathway was a response to the high level of TRIM44‐induced ICC cell EMT.

Clinically, we analyzed the TRIM44 expression in 130 ICC patients. We found that expression of TRIM44 was highly relevant with large tumor size, metastasis, poor tumor differentiation, and negatively related to patients' OS and recurrence. To conclude, we reveal that elevated TRIM44 promotes ICC progression by inducing ICC cell EMT and antiapoptosis, and TRIM44 serve as a poor prognosis marker for ICC.

## Conflict of Interest

Nothing need to be reported.

## Supporting information


**Figure S1.** Effect of TRIM44 in ICC cells proliferation, apoptosis, migration, and invasion.

**Figure S2.** Overexpressed TRIM44 promotes ICC cell invasiveness by inducing EMT.

**Figure S3.** Pathway in cell with high level of TRIM44.

**Data S1.** Method and Material.

**Table S1** List of primary antibodies used in the study.

**Table S2** sequence of primer for Real‐time polymerase chain reaction.
