# Supplementary figures and images for: Elevated TRIM44 promotes intrahepatic cholangiocarcinoma progression by inducing cell EMT via MAPK signaling
Source: Cancer Med. 2018 Feb 15;7(3):796–808. doi: 10.1002/cam4.1313 (PMC5852353; doi:10.1002/cam4.1313)

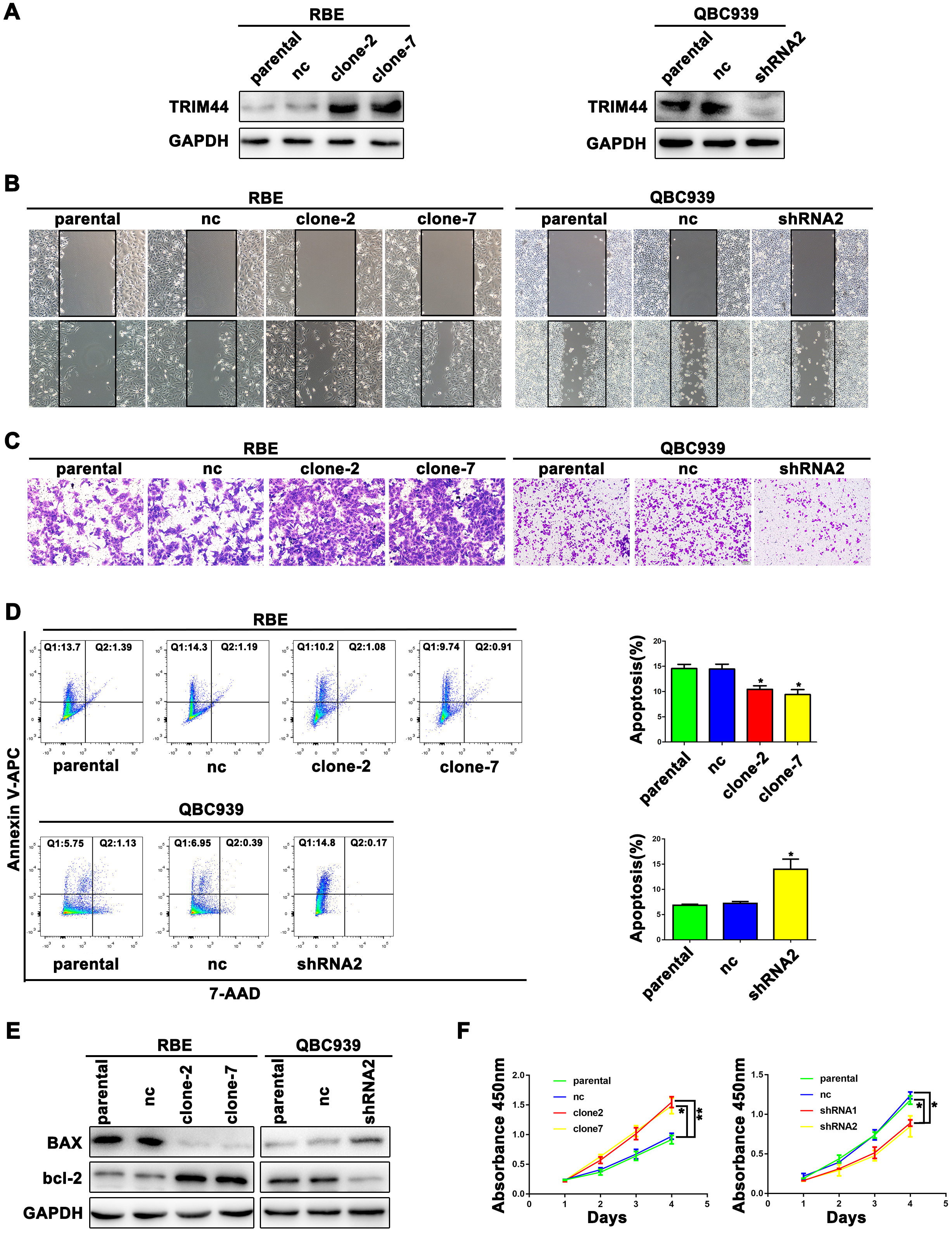

Supplement: Supplementary file 1 — Figure S1. Effect of TRIM44 in ICC cells proliferation, apoptosis, migration, and invasion. [file CAM4-7-796-s001.tif]

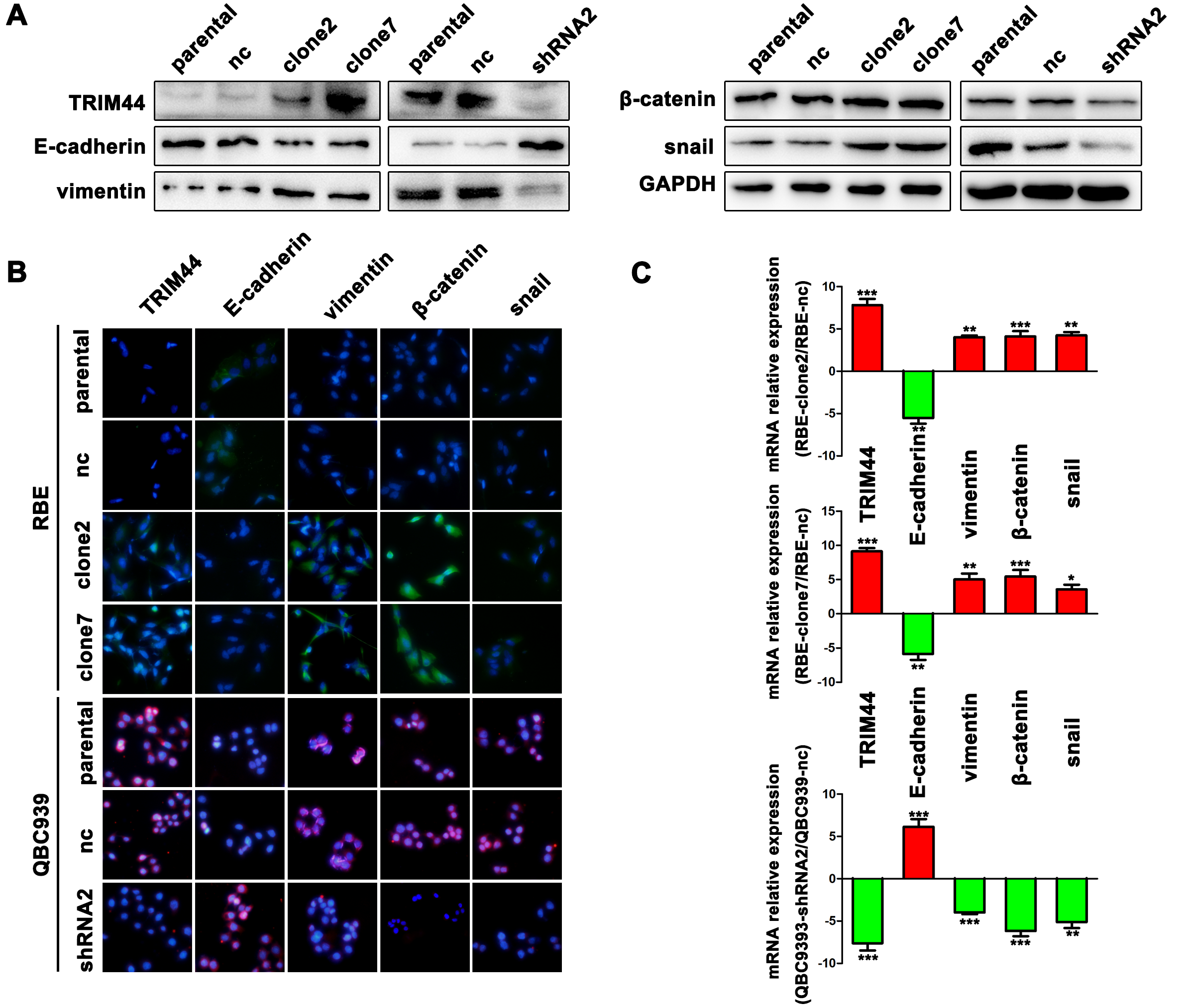

Supplement: Supplementary file 2 — Figure S2. Overexpressed TRIM44 promotes ICC cell invasiveness by inducing EMT. [file CAM4-7-796-s002.tif]

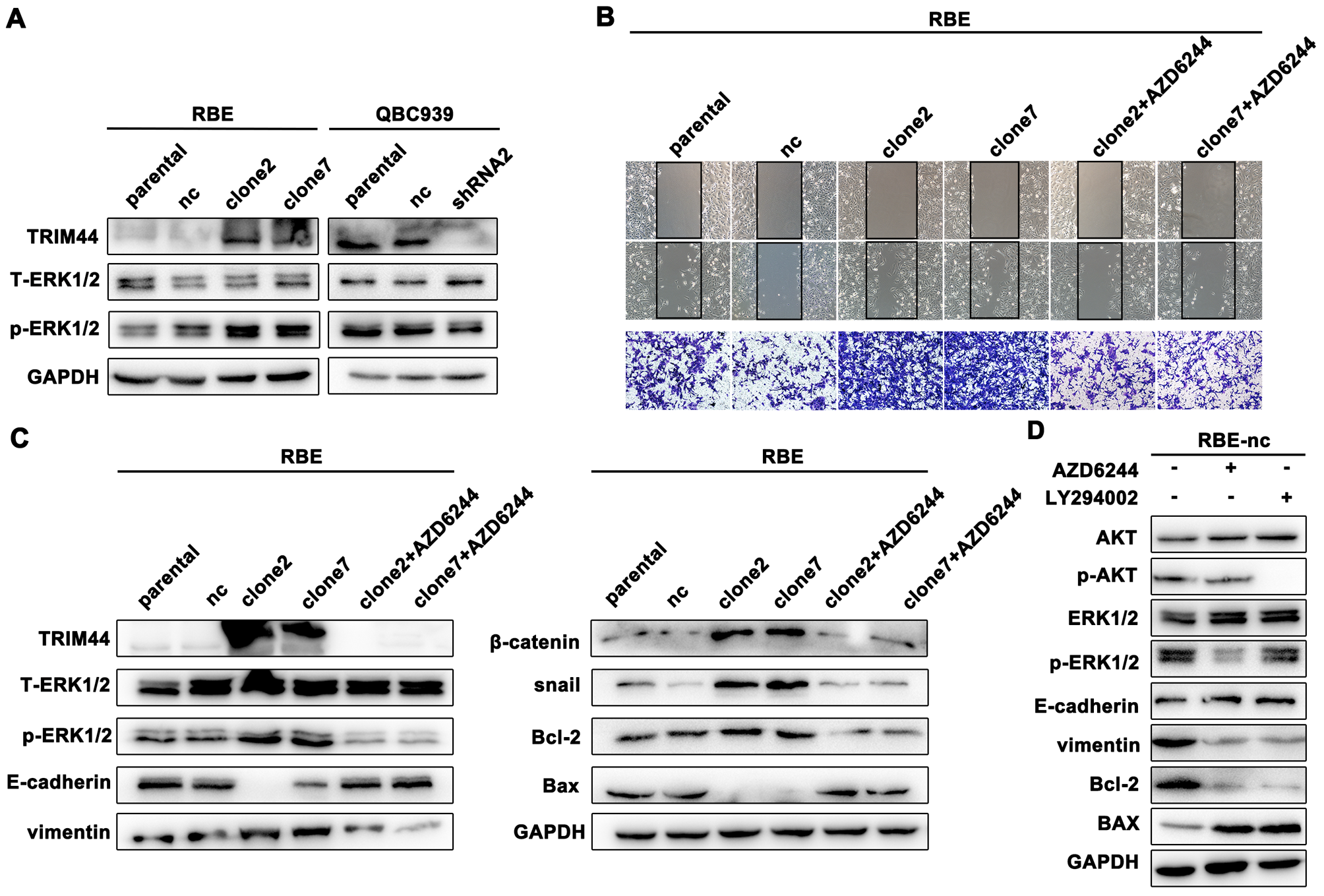

Supplement: Supplementary file 3 — Figure S3. Pathway in cell with high level of TRIM44. [file CAM4-7-796-s003.tif]
