# Supplementary material for: Elevated TRIM44 promotes intrahepatic cholangiocarcinoma progression by inducing cell EMT via MAPK signaling
Source: Cancer Med. 2018 Feb 15;7(3):796–808. doi: 10.1002/cam4.1313 (PMC5852353; doi:10.1002/cam4.1313)
Supplement: Supplementary file 4 — Data S1. Method and Material. [file CAM4-7-796-s004.docx]

**Supplementary Table 1** List of primary antibodies used in the study

| Antibody | Applications | Company |
| --- | --- | --- |
| TRIM44 | WB IHC | Proteintech(11511-1-AP) |
| TRIM44 | WB IF | Proteintech(66249-1-Ig) |
| GAPDH | WB | Cell signaling Technology |
| E-cadherin | WB IHC IF | Abcam |
| N-cadherin | WB | Abcam |
| slug | WB | Abcam |
| snail | WB IHC IF | Abcam |
| twist | WB | Abcam |
| β-catenin | WB IHC IF | Abcam |
| Bax | WB | Abcam |
| Bcl-2 | WB | Abcam |
| Caspase3 | WB | Affinity |
| Cleaved-caspase3 | WB | Affinity |
| Caspase9 | WB | Affinity |
| Cleaved-caspase9 | WB | Affinity |
| PRAP1 | WB | Affinity |
| Cleaved-PRAP1 | WB | Affinity |

**Abbreviations:** WB, western blot; IHC, immunohistochemistry; IF, immunofluorescence
