# Supplementary material for: Elevated TRIM44 promotes intrahepatic cholangiocarcinoma progression by inducing cell EMT via MAPK signaling
Source: Cancer Med. 2018 Feb 15;7(3):796–808. doi: 10.1002/cam4.1313 (PMC5852353; doi:10.1002/cam4.1313)
Supplement: Supplementary file 5 — Table S1 List of primary antibodies used in the study. [file CAM4-7-796-s005.docx]

**Supplementary Table 2** sequence of primer for Real-time polymerase chain reaction

| **TRIM44** |  |
| --- | --- |
| Forward | 5’AGGCAGCTCATCTGTGTCCT3’ |
| Reverse | 5’GCCTTCAGTCCACCTGAGTC3’ |
| **E-cadherin** |  |
| Forward | 5’GAACGCATTGCCACATACAC3’ |
| Reverse | 5’GAATTCGGGCTTGTTGTCAT3’ |
| **N-cadherin** |  |
| Forward | 5’AAACAGCAACGACGGGTTAG3’ |
| Reverse | 5’GTGCTGAATTCCCTTGGCTA3’ |
| **Vimentin** |  |
| Forward | 5’CCTTGACATTGAGATTGCCACCTA3’ |
| Reverse | 5’TCATCGTGATGCTGAGAAGTTTCG3’ |
| **β-catenin** |  |
| Forward | 5’GGAGCCCTTCACATCCTAGC3’ |
| Reverse | 5’AGCAGCTGCACAAACAATGG3’ |
| **Snail** |  |
| Forward | 5’TTTACCTTCCAGCAGCCCTA3’ |
| Reverse | 5’GGACAGAGTCCCAGATGAGC3’ |
| **Slug** |  |
| Forward | 5’CATCTTTGGGGCGAGTGAGTCC3’ |
| Reverse | 5’GGCCAGCCCAGAAAAAGTTGAAT3’ |
| **Twist** |  |
| Forward | 5’GTCCGCAGTCTTACGAGGAG3’ |
| Reverse | 5’GTCTGAATCTTGCTCAGCTTGTC3’ |
| **GAPDH** |  |
| Forward | 5’GGTATGACAACGAATTTGGC3’ |
| Reverse | 5’GAGCACAGGGTACTTTATTG3’ |
