# Supplementary material for: Elevated TRIM44 promotes intrahepatic cholangiocarcinoma progression by inducing cell EMT via MAPK signaling
Source: Cancer Med. 2018 Feb 15;7(3):796–808. doi: 10.1002/cam4.1313 (PMC5852353; doi:10.1002/cam4.1313)
Supplement: Supplementary file 6 — Table S2 sequence of primer for Real‐time polymerase chain reaction. [file CAM4-7-796-s006.docx]

**Supplementary Method and material**

**The criteria of selecting 130 ICC patients**

We randomly choose 130 patients who underwent curative resection at our hospital from 2007 to 2012, none of them had preoperative chemotherapy, and we did not include the patients who underwent only biopsy. Curative resection was defined as complete resection of tumor nodules, with the tumor margins rendered free of cancer on histologic examination, and resection of the regional lymph nodes, including the hilar, hepatoduodenal ligament lymph nodes and the caval lymph nodes, with no cancerous thrombus in the portal vein (main trunk or 2 major branches), hepatic veins, or bile duct. Patients with ICC who had lymph node involvement beyond these lymph nodes were categorized with distant metastasis and were excluded from the current study. The histopathologic diagnosis was based on World Health Organization criteria. Liver function was assessed by the Child-Pugh scoring system. The clinical classification of tumors was assigned according to the sixth edition of the TNM classification system published by the International Union Against Cancer. The histologic grade of tumor differentiation was determined according to the classification proposed by Edmondson and Steiner.

**The identifying of TRIM44 positive staining density**

Briefly, the density of positive staining was measured with a computerized image system composed of a Leica CCD camera DFC420 connected to a Leica DM IRE2 microscope (Leica Microsystems Imaging Solutions, Ltd., Cambridge, UK) blindly by two pathologists. Under high-power magnification (×200), photographs of four representative fields were captured by the Leica QWin Plus v3 software; identical settings were used for each photograph. The TRIM44 density was counted by Image-Pro Plus v6.2 software (Media Cybernetics, Inc., Bethesda, MD). For the reading of each antibody staining, a uniform setting for all the slides was applied. Integrated optical density (IOD) of all positive staining of TRIM44 in each photograph was measured and its ratio to total area of each photograph was calculated as TRIM44 density.
