# Supplementary material for: Elevated TRIM44 promotes intrahepatic cholangiocarcinoma progression by inducing cell EMT via MAPK signaling
Source: Cancer Med. 2018 Feb 15;7(3):796–808. doi: 10.1002/cam4.1313 (PMC5852353; doi:10.1002/cam4.1313)
Supplement: Supplementary file 7 [file CAM4-7-796-s007.docx]

**Supplementary Figure Legends**

**Supplementary Figure 1. Effect of TRIM44 in ICC cells proliferation, apoptosis, migration, and invasion**

**A.** Regulation of TRIM44 expression in RBE and QBC939. Stable clone 2 and stable clone 7 were chosen from the RBE cell which treated with TRIM44 overexpressed vectors 72h, and confirmed by western blotting. **B.** Wound healing assays showed the TRIM44 expression positively associated with the ICC cell migration. **C.** The invasion of seven cell lines were measured by transwell assays. **D.** The apoptosis of two RBE-clones, QBC939-shRNA2 and its control cells were analyzed by FACS. The quadrant of Q1 means early apoptotic cells sub-populations. The quadrant of Q2 means late stage apoptotic cells sub-populations. **E.** Western blot indicated that TRIM44 induced the level of Bax, Bcl-2, caspase3, caspase9 and PARP. GAPDH was used as internal control. **F.** CCK-8 assays illustrated that high level of TRIM44 can promote the cell viability; OD indicates optical density. *p<0.05, **p<0.01.

**Supplementary Figure 2. Overexpressed TRIM44 promotes ICC cell invasiveness by inducing EMT.**

**A and B.** The mRNA and protein of EMT markers were compared between five cell lines (RBE-nc, RBE-clone2, RBE-clone7, QBC939-nc, QBC939-shRNA2). GAPDH is internal control. **C.** Immunofluoresce of TRIM44, E-cadherin, vimentin, β-catenin and snail in ICC cell lines. *p<0.05, **p<0.01, ***p<0.001.

**Supplementary Figure 3. Pathway in cell with high level of TRIM44**

**A.** MAPK-ERK1/2 pathway were observed in RBE-clone2 and clone7, QBC939-shRNA2 and their control cells. **B.** Wound healing and invasion assays compared with parental cell, RBE-nc, RBE-clone2, clone-7 with PBS treatment and RBE-clones treated by AZD6244 (MEK inhibitor, 5μM, 24h). **C.** Western blot determined TRIM44, ERK1/2, p-ERK1/2, EMT markers and transcription factors. GAPDH was used as internal control. **D.** Western blot showed that AKT, ERK1/2 and several proteins expression in RBE-nc cells which were incubated with MEK inhibitor and AKT inhibitor
